# Supplementary material for: NGF-TrkA signaling dictates neural ingrowth and aberrant osteochondral differentiation after soft tissue trauma
Source: Nat Commun. 2021 Aug 16;12:4939. doi: 10.1038/s41467-021-25143-z (PMC8368242; doi:10.1038/s41467-021-25143-z)
Supplement: Supplementary file 3 — Reporting Summary [file 41467_2021_25143_MOESM3_ESM.pdf]

nature research

Corresponding author(s): Benjamin Levi, M.D. & Aaron James, M.D., Ph.D.

Last updated by author(s): May 11, 2021

Reporting Summary

Nature Research wishes to improve the reproducibility of the work that we publish. This form provides structure for consistency and transparency in reporting. For further information on Nature Research policies, see our Editorial Policies and the Editorial Policy Checklist.

Statistics

For all statistical analyses, confirm that the following items are present in the figure legend, table legend, main text, or Methods section.

n/a	Confirmed	
☐	☒	The exact sample size (n) for each experimental group/condition, given as a discrete number and unit of measurement
☐	☒	A statement on whether measurements were taken from distinct samples or whether the same sample was measured repeatedly
☐	☒	The statistical test(s) used AND whether they are one- or two-sided<br>*Only common tests should be described solely by name; describe more complex techniques in the Methods section.*
☐	☒	A description of all covariates tested
☐	☒	A description of any assumptions or corrections, such as tests of normality and adjustment for multiple comparisons
☐	☒	A full description of the statistical parameters including central tendency (e.g. means) or other basic estimates (e.g. regression coefficient) AND variation (e.g. standard deviation) or associated estimates of uncertainty (e.g. confidence intervals)
☐	☒	For null hypothesis testing, the test statistic (e.g. F, t, r) with confidence intervals, effect sizes, degrees of freedom and P value noted<br>*Give P values as exact values whenever suitable.*
☒	☐	For Bayesian analysis, information on the choice of priors and Markov chain Monte Carlo settings
☒	☐	For hierarchical and complex designs, identification of the appropriate level for tests and full reporting of outcomes
☐	☒	Estimates of effect sizes (e.g. Cohen's d, Pearson's r), indicating how they were calculated

Our web collection on statistics for biologists contains articles on many of the points above.

Software and code

Policy information about availability of computer code

Data collection	Cell Ranger Single Cell Software Suite 1.3<br>10X Genomic software Cell Ranger
Data analysis	FlowJo Software v10<br>Imaris Software v9.3<br>Osteomeasure Software

For manuscripts utilizing custom algorithms or software that are central to the research but not yet described in published literature, software must be made available to editors and reviewers. We strongly encourage code deposition in a community repository (e.g. GitHub). See the Nature Research guidelines for submitting code & software for further information.

Data

Policy information about availability of data

All manuscripts must include a data availability statement. This statement should provide the following information, where applicable:
- Accession codes, unique identifiers, or web links for publicly available datasets
- A list of figures that have associated raw data
- A description of any restrictions on data availability

Sequencing data is available through NCBI Gene Expression Omnibus at accession numbers ‘GSE126060 [https://www.ncbi.nlm.nih.gov/geo/query/acc.cgi?acc=GSE126060]’ and ‘GSE163446 [https://www.ncbi.nlm.nih.gov/geo/query/acc.cgi?acc=GSE163446]’. Microarray data from human spinal ligament cells was obtained from publicly available Gene Expression Omnibus (GEO) dataset ‘GSE5464 [https://www.ncbi.nlm.nih.gov/geo/query/acc.cgi?acc=GSE5464]’. Source data are provided with this paper. All other relevant data supporting the findings of this study are included within the article and its Supplementary Materials files or from the corresponding authors upon reasonable request.

Field-specific reporting

Please select the one below that is the best fit for your research. If you are not sure, read the appropriate sections before making your selection.

- [x] Life sciences
- [ ] Behavioural & social sciences
- [ ] Ecological, evolutionary & environmental sciences

For a reference copy of the document with all sections, see nature.com/documents/nr-reporting-summary-flat.pdf

Life sciences study design

All studies must disclose on these points even when the disclosure is negative.

Sample size	All sample size calculation was performed by using G*Power version 3.1.9.2 (Franz Faul, Universitat Kiel, Germany). Sample size calculations were performed based on an anticipated effect size range of 2.66 - 3.75, using our previously published data in adult mice. For this scenario, with three samples per group, a two-sample t-test would provide 80% power to detect effect sizes of at least 2.0 assuming a two-sided 0.05 level of significance.
Data exclusions	No data were excluded from the data analyses
Replication	A total of ~4 billion reads were generated from the 10X Genomics sequencing analysis for fifteen replicates (4 replicates for days 0, 7, and 21; 3 replicates for day 3). All replication attempts were successful. The sequencing data was first pre-processed using the 10X Genomics software Cell Ranger (10x Genomics Inc., Pleasanton, CA, USA) and aligned to mm10 genome. Downstream analysis steps were performed using Seurat. Each replicate was initially considered independently.
Randomization	Mice were randomly assigned to treatment groups whenever feasible (for data presented in Figure 5 and 7). A Shapiro-Wilk test for normality was performed on all datasets. Homogeneity was confirmed by a comparison of variances test.
Blinding	Blinding was performed during data collection and analyses. In some experimental settings (histological analysis of Fig 2,5,6,7), the differences in histology were so obvious between groups so as to lead to unintentional unblinding during the course of analyses. MicroCT scans were analyzed by blinded operators manually splining around ectopic tissue.

Reporting for specific materials, systems and methods

We require information from authors about some types of materials, experimental systems and methods used in many studies. Here, indicate whether each material, system or method listed is relevant to your study. If you are not sure if a list item applies to your research, read the appropriate section before selecting a response.

Materials & experimental systems

n/a	Involved in the study	
☐	☒	Antibodies
☒	☐	Eukaryotic cell lines
☒	☐	Palaeontology and archaeology
☐	☒	Animals and other organisms
☐	☒	Human research participants
☒	☐	Clinical data
☒	☐	Dual use research of concern

Methods

n/a	Involved in the study	
☒	☐	ChIP-seq
☐	☒	Flow cytometry
☒	☐	MRI-based neuroimaging

Antibodies

Antibodies used

Name Vendor Catalog No. Concentration Use
Anti-Aggrecan Abcam ab3778 1:200 IF
Anti-CD11b BD Biosciences 562950 1:100 F
Anti-CD45 BioLegend 103144 1:200 IF
Anti-CGRP Sigma Aldrich C8198 1:200 IF
Anti-Col2 Abcam ab185430 1:200 IF
Anti-ColX Abcam ab58632 1:200 IF
Anti-FGF2 Santa Cruz sc-365106 1:100 IF
Anti-F4/80 Abcam ab204467 1:200 IF
Anti-F4/80 BD Biosciences 746070 1:100 F
Anti-Ly6C BD Biosciences 553104 1:50 F
Anti-Ly6G BD Biosciences 551459 1:100 F
Anti-NGF Abcam ab6199 1:100 IHC
Anti-NGF Abcam ab52918 1:100 WB
Anti-pro-NGF Santa Cruz 365944 1:100 WB

Anti-Osteocalcin Abcam ab93876 1:200 IF
Anti-PDGFRA Abcam ab15501 N/A IF
Anti-PDGFRA Abcam ab96569 1:100 IHC
Anti-pERK1/2 Cell Signaling 9101S 1:100 IF
Anti-pTrkA Invitrogen PA5-37672 1:200 IF
Anti-PGP9.5 Agilent Tech. Z511601-2 1:200 IF
Anti-pSmad2 Invitrogen 44-244G 1:100 IF
Anti-S100 Roche Diagnostics 790-2914 N/A IHC
Anti-SM31 EMD Millipore NE1022 1:20,000 IHC
Anti-SMA Abcam ab7817 1:200 IF
Anti-SOX9 Abcam ab185230 1:200 IF
Anti-TH EMD Millipore AB152 1:200 IF
Anti-TNMD Abcam ab203676 1:200 IF
Anti-TUBB3 Abcam ab18207 1:1500 IF
Anti-Mouse IgG Vector MP-5402 1:200 IHC
Anti-rabbit IgG, HRP Cell Signaling 7074S 1:200 WB
GAPDH Cell Signaling 51745 1:1000 WB
Goat Anti-Mouse IgG Abcam ab150119 1:200 IF
Goat Anti-Rabbit IgG Vector DI-1594 1:200 IF

Validation

1. Anti-Aggrecan Abcam ab3778 1:200 IF Li L et al. XIST/miR-376c-5p/OPN axis modulates the influence of proinflammatory M1 macrophages on osteoarthritis chondrocyte apoptosis. J Cell Physiol 235:281-293 (2020).
2. Anti-CD11b BD Biosciences 562950 1:100 F Kaji K, Takeshita S, Miyake K, Takai T, Kudo A. Functional association of CD9 with the Fc gamma receptors in macrophages. J Immunol. 2001; 166(5):3256-3265.
3. Anti-CD45 BioLegend 103144 1:200 IF Li Z, Meyers CA, Chang L, et al. Fracture repair requires TrkA signaling by skeletal sensory nerves. J Clin Invest. 2019;129(12):5137-5150.
4. Anti-CGRP Sigma Aldrich C8198 1:200 IF Südhof TC. The synaptic vesicle cycle: a cascade of protein-protein interactions. Nature. 1995 Jun 22;375(6533):645-53.
5. Anti-Col2 Abcam ab185430 1:200 IF Lin C, Liu L, Zeng C, Cui ZK, Chen Y, Lai P, Wang H, Shao Y, Zhang H, Zhang R, Zhao C, Fang H, Cai D, Bai X. Activation of mTORC1 in subchondral bone preosteoblasts promotes osteoarthritis by stimulating bone sclerosis and secretion of CXCL12. Bone Res. 2019 Feb 20;7:5.
6. Anti-ColX Abcam ab58632 1:200 IF Yu L et al. BMP9 stimulates joint regeneration at digit amputation wounds in mice. Nat Commun 10:424 (2019).
7. Anti-FGF2 Santa Cruz sc-365106 1:100 IF Aziz K, Sieben CJ, Jeganathan KB, Hamada M, Davies BA, Velasco ROF, Rahman N, Katzmann DJ, van Deursen JM. Mosaic-variegated aneuploidy syndrome mutation or haploinsufficiency in Cep57 impairs tumor suppression. J Clin Invest. 2018 Aug 1;128(8):3517-3534.
8. Anti-F4/80 Abcam ab204467 1:200 IF Kopecky C, Pandzic E, Parmar A, Szajer J, Lee V, Dupuy A, Arthur A, Fok S, Whan R, Ryder WJ, Rye KA, Cochran BJ. Translocator protein localises to CD11b+ macrophages in atherosclerosis. Atherosclerosis. 2019 May;284:153-159.
9. Anti-F4/80 BD Biosciences 746070 1:100 F Bodhankar S, Lapato A, Chen Y, Vandenbark AA, Saugstad JA, Offner H. Role for microglia in sex differences after ischemic stroke: importance of M2. Metab Brain Dis. 2015 Dec;30(6):1515-29.
10. Anti-Ly6C BD Biosciences 553104 1:50 F Wrammert J, Källberg E, Agace WW, Leanderson T. Ly6C expression differentiates plasma cells from other B cell subsets in mice. Eur J Immunol. 2002 Jan;32(1):97-103.
11. Anti-Ly6G BD Biosciences 551459 1:100 F Fleming TJ, Malek TR. Multiple glycosylphosphatidylinositol-anchored Ly-6 molecules and transmembrane Ly-6E mediate inhibition of IL-2 production. J Immunol. 1994 Sep 1;153(5):1955-62.
12. Anti-NGF Abcam ab6199 1:100 IHC Huang J, Zhao L, Fan Y, Liao L, Ma PX, Xiao G, Chen D. The microRNAs miR-204 and miR-211 maintain joint homeostasis and protect against osteoarthritis progression. Nat Commun. 2019 Jun 28;10(1):2876.
13. Anti-NGF Abcam ab52918 1:100 WB Chen H, Zhang J, Dai Y, Xu J. Nerve growth factor inhibits TLR3-induced inflammatory cascades in human corneal epithelial cells. J Inflamm (Lond). 2019 Dec 26;16:27.
14. Anti-pro-NGF Santa Cruz 365944 1:100 WB Rafa-Zabłocka K, Kreiner G, Bagińska M, Nalepa I. Selective Depletion of CREB in Serotonergic Neurons Affects the Upregulation of Brain-Derived Neurotrophic Factor Evoked by Chronic Fluoxetine Treatment. Front Neurosci. 2018 Sep 20;12:637.
15. Anti-Osteocalcin Abcam ab93876 1:200 IF Wang X et al. Inhibition of overactive TGF-ß attenuates progression of heterotopic ossification in mice. Nat Commun 9:551 (2018).
16. Anti-PDGFRA Abcam ab15501 N/A IF Taniguchi, E., Nishijo, K., McCleish, A. et al. PDGFR-A is a therapeutic target in alveolar rhabdomyosarcoma. Oncogene 27, 6550–6560 (2008). https://doi.org/10.1038/onc.2008.255
17. Anti-PDGFRA Abcam ab96569 1:100 IHC Debnath S et al. Discovery of a periosteal stem cell mediating intramembranous bone formation. Nature 562:133-139 (2018).
18. Anti-pERK1/2 Cell Signaling 9101S 1:100 IF Li Y, Li B, Li W, Wang Y, Akgül S, Treisman DM, Heist KA, Pierce BR, Hoff B, Ho CY, Ferguson DO, Rehemtulla A, Zheng S, Ross BD, Li JZ, Zhu Y. Murine models of IDH-wild-type glioblastoma exhibit spatial segregation of tumor initiation and manifestation during evolution. Nat Commun. 2020 Jul 22;11(1):3669.
19. Anti-pTrkA Invitrogen PA5-37672 1:200 IF Species: Human, Mouse, Rat / Expression System: Rabbit, IgG / Class: Polyclonal / Type: Antibody / Immunogen: Peptide sequence around phosphorylation site of tyrosine791 (P-V-Y(p)-L-D) derived from Human TrkA / Conjugate: Unconjugated
20. Anti-PGP9.5 Agilent Tech. Z511601-2 1:200 IF Meyers, C., Lee, S., Sono, T., Xu, J., Negri, S., Tian, Y., Wang, Y., Li, Z., Miller, S., Chang, L., Gao, Y., Minichiello, L., Clemens, T., & James, A. (2020). A Neurotrophic Mechanism Directs Sensory Nerve Transit in Cranial Bone. Cell reports, 31, 107696 - 107696.
21. Anti-pSmad2 Invitrogen 44-244G 1:100 IF Ruiz-Gutierrez M, Bölükbaşı ÖV, Alexe G, Kotini AG, Ballotti K, Joyce CE, Russell DW, Stegmaier K, Myers K, Novina CD, Papapetrou EP, Shimamura A. Therapeutic discovery for marrow failure with MDS predisposition using pluripotent stem cells. JCI Insight. 2019 Apr 30;5(12):e125157.
22. Anti-S100 Roche Diagnostics 790-2914 N/A IHC This antibody is intended for in vitro diagnostic (IVD) use. Ventana Medical

Systems, Inc. (Ventana) CONFIRM anti-S100 (4C4.9) Primary Antibody is a mouse monoclonal antibody (IgG2a) directed against S100 protein. This antibody is intended for use to qualitatively identify S100 protein by light microscopy in sections of formalin fixed, paraffin embedded tissue on a Ventana automated slide stainer. The clinical interpretation of any staining, or the absence of staining, must be complemented by morphological studies and evaluation of proper controls. Evaluation must be made by a qualified pathologist within the context of the patient's clinical history and other diagnostic tests.
23. Anti-SM31 EMD Millipore NE1022 1:20,000 IHC Yang CC, Alvarez RB, Engel WK, Heller SL, Askanas V. Nitric oxide-induced oxidative stress in autosomal recessive and dominant inclusion-body myopathies. Brain. 1998 Jun;121 (Pt 6):1089-97.
24. Anti-SMA Abcam ab7817 1:200 IF Pein M et al. Metastasis-initiating cells induce and exploit a fibroblast niche to fuel malignant colonization of the lungs. Nat Commun 11:1494 (2020).
25. Anti-SOX9 Abcam ab185230 1:200 IF Ichimaru S, Nakagawa S, Arai Y, et al. Hypoxia Potentiates Anabolic Effects of Exogenous Hyaluronic Acid in Rat Articular Cartilage. Int J Mol Sci. 2016;17(7):1013.
26. Anti-TH EMD Millipore AB152 1:200 IF Edri R, Yaffe Y, Ziller MJ, Mutukula N, Volkman R, David E, Jacob-Hirsch J, Malcov H, Levy C, Rechavi G, Gat-Viks I, Meissner A, Elkabetz Y. Analysing human neural stem cell ontogeny by consecutive isolation of Notch active neural progenitors. Nat Commun. 2015 Mar 23;6:6500.
27. Anti-TNMD Abcam ab203676 1:200 IF Li C, Wang N, Schäffer AA, Liu X, Zhao Z, Elliott G, Garrett L, Choi NT, Wang Y, Wang Y, Wang C, Wang J, Chan D, Su P, Cui S, Yang Y, Gao B. Mutations in COMP cause familial carpal tunnel syndrome. Nat Commun. 2020 Jul 20;11(1):3642.
28. Anti-TUBB3 Abcam ab18207 1:1500 IF Baral P, Umans BD, Li L, Wallrapp A, Bist M, Kirschbaum T, Wei Y, Zhou Y, Kuchroo VK, Burkett PR, Yipp BG, Liberles SD, Chiu IM. Nociceptor sensory neurons suppress neutrophil and γδ T cell responses in bacterial lung infections and lethal pneumonia. Nat Med. 2018 May;24(4):417-426.
29. Anti-Mouse IgG Vector MP-5402 1:200 IHC Sorkin, M., Huber, A.K., Hwang, C. et al. Regulation of heterotopic ossification by monocytes in a mouse model of aberrant wound healing. Nat Commun 11, 722 (2020).
30. Anti-rabbit IgG, HRP Cell Signaling 7074S 1:200 WB Abboud D, Daly AF, Dupuis N, Bahri MA, Inoue A, Chevigné A, Ectors F, Plenevaux A, Pirotte B, Beckers A, Hanson J. GPR101 drives growth hormone hypersecretion and gigantism in mice via constitutive activation of Gs and Gq/11. Nat Commun. 2020 Sep 21;11(1):4752.
31. GAPDH Cell Signaling 5174S 1:1000 WB Kang L, Yu H, Yang X, Zhu Y, Bai X, Wang R, Cao Y, Xu H, Luo H, Lu L, Shi MJ, Tian Y, Fan W, Zhao BQ. Neutrophil extracellular traps released by neutrophils impair revascularization and vascular remodeling after stroke. Nat Commun. 2020 May 19;11(1):2488.
32. Goat Anti-Mouse IgG Abcam ab150119 1:200 IF Shukla S et al. Inhibition of telomerase RNA decay rescues telomerase deficiency caused by dyskerin or PARN defects. Nat Struct Mol Biol 23:286-92 (2016).
33. Goat Anti-Rabbit IgG Vector DI-1594 1:200 IF Timothy S. Jarvela, Hoa A. Lam, Michael Helwig, Nikolai Lorenzen, Daniel E. Otzen, Pamela J. McLean, Nigel T. Maidment, Iris Lindberg. Proceedings of the National Academy of Sciences Aug 2016, 113 (32) E4708-E4715

Animals and other organisms

Policy information about studies involving animals; ARRIVE guidelines recommended for reporting animal research

Laboratory animals	Male and Female, 6-8 weeks old, C57BL/6J Jackson Laboratory, Stock #000664<br>Male and Female, 6-8 weeks old, mT/mG Jackson Laboratory, Stock #007576<br>Male and Female, 6-8 weeks old, NGF-eGFP Donated from Kawaja laboratory<br>Male and Female, 6-8 weeks old, Ngf fl/fl Donated from Minichiello laboratory<br>Male and Female, 6-8 weeks old, TrkAF592A Donated from Ginty laboratory, Jackson Laboratory, Stock #022362<br><br>All animals were housed in IACUC-supervised facilities at 18°C–22°C, 50% (±20%) of relative humidity, 12-hr light-dark cycle with ad libitum access to food and water.
Wild animals	The study did not involve wild animals
Field-collected samples	The study did not involve samples collected from the field
Ethics oversight	All animal procedures were complied with all relevant ethical regulations for animal testing and research, and were carried out in accordance with the guidelines provided in the Guide for the Use and Care of Laboratory Animals from the Institute for Laboratory Animal Research (ILAR, 2011) and were approved by the Institutional Animal Care and Use Committee (IACUC) of the University of Michigan (PRO0007390) or Johns Hopkins University (MO16M226 & MO19M366).

Note that full information on the approval of the study protocol must also be provided in the manuscript.

Human research participants

Policy information about studies involving human research participants

Population characteristics	Pathology samples were obtained from patients with a mean age of 33.3 years, and were 50% male and 50% female.
Recruitment	Subjects were not recruited for this pathology analysis.
Ethics oversight	Johns Hopkins University Institutional Review Board approval

Note that full information on the approval of the study protocol must also be provided in the manuscript.

Flow Cytometry

Plots

Confirm that:

- [x] The axis labels state the marker and fluorochrome used (e.g. CD4-FITC).
- [x] The axis scales are clearly visible. Include numbers along axes only for bottom left plot of group (a 'group' is an analysis of identical markers).
- [x] All plots are contour plots with outliers or pseudocolor plots.
- [x] A numerical value for number of cells or percentage (with statistics) is provided.

Methodology

Sample preparation	After HO induction surgery, the soft tissue around the injury site was dissected from the posterior compartment between the muscular origin and calcaneal insertion of Achilles tendon at the indicated time points. Tissue was digested for 45 min in 0.3% Type 1 Collagenase and 0.4% Dispase II (Gibco) in Roswell Park Memorial Institute (RPMI) medium at 37°C under constant agitation at 120rpm. Digestions were subsequently quenched with 10% FBS RPMI and filtered through 40µm sterile strainers. Specimens were blocked with anti-mouse CD16/32 and subsequently stained using the following antibodies: FITC:Ly6C, BV510:CD11b, APCH7:Ly6G, and BB700:F4/80.
Instrument	FACSAria II for cell sorting or LSRFortessa for analysis (BD)
Software	FlowJo software.
Cell population abundance	Cell population abundance was measured among CD45, CD11b, Ly6G, Ly6C, and F4/80 cells, as determined in triplicate and using the below gating strategy.
Gating strategy	Cells were gated based on FSC vs SSC. After this, doublet exclusion was performed both by comparing FSC-height vs FSC-width and SSC-height vs. SSC-width. Propidium Iodide staining determined the live cells and all negative stained cells were gated. Once this was determined, CD11b+ cells was gated. These CD11b cells were then analyzed their expression of granulocyte and myeloid cell markers, Ly6G and Ly6C respectively and gated into Ly6Chi monocytes, ly6Clo monocytes, Ly6C and Ly6G double negative macrophages, and Ly6G positive neutrophils. Macrophages were further analyzed for their expression of macrophage marker F4/80. Unstained controls were used to determine the negative gate.

- [x] Tick this box to confirm that a figure exemplifying the gating strategy is provided in the Supplementary Information.
