# Supplementary material for: NGF-TrkA signaling dictates neural ingrowth and aberrant osteochondral differentiation after soft tissue trauma
Source: Nat Commun. 2021 Aug 16;12:4939. doi: 10.1038/s41467-021-25143-z (PMC8368242; doi:10.1038/s41467-021-25143-z)
Supplement: Supplementary file 1 — Supplementary Information [file 41467_2021_25143_MOESM1_ESM.docx]

**Supplementary Materials**

**Supplementary Figures and Figure Legends**

**Supplementary Fig. S1**

Supplementary Fig. S1: Validation of hypoalgesia with surgical denervation or TrkA inhibition. (a) von Frey testing at 1 wk post-injury with or without sciatic neurectomy in comparison to uninjured control (* *p*=0.035, ** *p*=0.008). (b) von Frey testing among TrkA^WT^ or TrkA^F592A^ animals, analyzed at 1 wk post-injury in comparison to uninjured control (*** *p*=0.0002, *** *p*=0.0001). For all graphs, each dot represents a single animal, with mean ± 1 SD indicated. N=6 animals per group, except for Sham 1 week post denervation (N=5 animals). Statistical analysis performed using a two-sided one-way ANOVA with post-hoc Tukey’s test. Source data are provided as a Source Data file.

**Supplementary Fig. S2**

Supplementary Fig. S2. Minor changes in animal activity after HO induction with or without sciatic neurectomy. Quantitative behavioral analysis assessed by video based tracking (ANYmaze) of open field activity among animals after HO induction with or without proximal sciatic neurectomy. Animals were subjected to HO induction, with or without concomitant proximal sciatic neurectomy, and examined at 3 and 7 days post-injury. For all graphs, each dot represents a single animal, with mean ± 1 SD indicated; N=5 animals per group. (a) Distance travelled, * *p*=0.050 vs. Sham. (b) Average speed, * *p*=0.042 vs. Sham. (c) Total time in motion, Day 3, * *p*=0.039 vs. Sham; Day 7, * *p*=0.049 vs. Sham. (d) total time stationary, Day 3, * *p*=0.042 vs. Sham; Day 7, * *p*=0.048 vs. Sham. (e) Mobile episodes, * *p*=0.011 vs. Sham. (f) Immobile episodes, * *p*=0.0101 vs. Sham. (g) Maximum speed, * *p*=0.044 vs. Sham. (h) Bodily rotation, * *p*=0.036 vs. Sham. (l) Latency to start of first mobile episode, * *p*=0.050 vs. Sham. Statistical analysis performed using a two-sided one-way ANOVA. Source data are provided as a Source Data file.

**Supplementary Fig. S3**

Supplementary Fig. S3. Validation of single cell clusters within trauma-induced heterotopic ossification. Cells at the injury site were collected prior to surgery (day 0) and at 3, 7 and 21 days after HO induction. (a) t-SNE plots of 9 cell clusters across timepoints. (b) Violin plots to demonstrate characteristic gene markers across cell populations including *Lyve1* (endothelial lymphatic), *Lyz2* (mixed inflammatory), *Irf8* (T cell), *Cd19* (B cell), *Csf3r* (neutrophil) and *Myf5* (skeletal muscle precursor). Characteristic genes for clusters 1, 2, and 3 are shown in Fig. 3. Cells isolated from N=4 animals for days 0, 7, and 21; N=3 animals for day 3.

**Supplementary Fig. S4**

Supplementary Fig. S4. Neurotrophic receptor tyrosine kinase 1 (*Ntrk1*) and Nerve growth factor receptor (*Ngfr*) expression within trauma-induced heterotopic ossification. Cells at the injury site were collected prior to injury (day 0) and at 3, 7 and 21 days after HO induction. (a) *Ntrk1* expression within t-SNE plots, shown across each timepoint individually. (b) *Ntrk1* expression within violin plots of 9 cell clusters, merged across all timepoints. (c) *Ngfr* expression within t-SNE plots, shown across each timepoint individually. (d) *Ngfr* expression within violin plots of 9 cell clusters, merged across all timepoints. Cells isolated from N=4 animals for days 0, 7, and 21; N=3 animals for day 3.

**Supplementary Fig. S5**

Supplementary Fig. S5. Protein expression of NGF and pro-NGF at the injury site. (a) NGF-eGFP reporter activity at the tenotomy site following 1 week post-injury. (b) Immunohistochemistry for NGF within the same image. (c) Merged images of (a,b). Dashed lines indicate margins of tendon. (d) NGF and pro-NGF at the injury site at days 0, 3, and 7 by western blot. (e) Quantification of NGF (* *p*=0.026). (f) Quantification of proNGF († *p*=0.052, * *p*=0.042). (g) ELISA based measurement of pro-NGF expression at the HO site, days 0, 3 and 7-post injury (* *p*=0.032, ** *p*=0.004). Scale bar: 100µm. Statistical analysis performed using a two-sided one-way ANOVA with post-hoc Tukey’s test. Data presented as a Mean ± 1 SD, with individual datapoints shown. N=3 animals per group for all assays. Source data are provided as a Source Data file.

**Supplementary Fig. S6**

Supplementary Fig. S6. pTrkA within the ipsilateral lumbar DRG following HO-inducing injury. (a) Immunohistochemistry for pTrkA in the DRGs among uninjured and (b) injured mice at 2 days post-HO inducing injury. (c) Quantification of (a,b). Scale bar: 100 µm. Each dot represents an individual animal, with mean ± 1 SD indicated. Statistical analysis performed using a two-way Student’s *t*-test. N=4 animals per group. *** *p*=0.0001. Source data are provided as a Source Data file.

**Supplementary Fig. S7**

Supplementary Fig. S7. Systemic distribution of Ad-GFP following injury site injection. Immunofluorescent images demonstrate that GFP reporter activity was seen in multiple tissues, including the (a) ipsilateral L5 DRG, (b) spleen, (c) kidney, (d) liver, (e) lung, and (f) heart. Each experiment was repeated three times independently with similar results. Scale bar: 50 µm.

**Supplementary Fig. S8**

Supplementary Fig. S8. Vascularity of the HO site with *Ngf* deletion. Vascularity was assessed among the injury site of *Ngf* floxed animals treated with Ad-GFP or Ad-Cre. (a,b) CD31 immunofluorescent staining of the distal tenotomy site, shown in sagittal cross-section. Dashed white line indicates the tendon edge. (c) Quantification of (a,b). Scale bar: 100 µm. Each dot represents an individual animal, with mean ± 1 SD indicated. Statistical analysis performed using a two-way Student’s *t*-test. N=5 animals per group. *** *p*=0.0002. Source data are provided as a Source Data file.

**Supplementary Fig. S9**

Supplementary Fig. S9. Lack of tendon phenotype in 1NMPP1-treated TrkA^F592A^ animals. Analysis of uninjured Achilles tendon among TrkA^WT^ and TrkA^F592A^ mice treated with 1NMPP1 for 9 wks. (a,b) H&E appearance of uninjured tendon among 1NMPP1-treated TrkA^WT^ and TrkA^F592A^ mice. (c) Mean tendon width was assessed using serial histologic cross-sections. For all graphs, each dot represents a single animal, with mean ± 1 SD indicated; N=5 per group. No statistical differences observed, as determined by a two-way Student’s *t*-test. Source data are provided as a Source Data file.

**Supplementary Fig. S10**

Supplementary Fig. S10. Loss of nerve density within the uninjured Achilles tendon following 1NMPP1 treatment in TrkA^F592A^ animals. Analysis of uninjured Achilles tendon among TrkA^F592A^ untreated with 1NMPP1 and treated with 1NMPP1 for 9 wks. (a,b) Beta Tubulin 3 (TUBB3) ^+^ immunofluorescent staining of the uninjured tendon among 1NMPP1-untreated TrkA^F592A^ and 1NMPP1-treated TrkA^F592A^ mice, as visualized using sagittal cross-sections. Dashed white lines indicate edges of Achilles tendon. (c) Quantified TUBB3^+^ nerve density among 1NMPP1-untreated TrkA^F592A^ and 1NMPP1-treated TrkA^F592A^ mice. Each dot represents a single animal, with mean ± 1 SD indicated; N=3 animals for TrkA^F592A^ without 1NMPP1 and N=5 animals for TrkA^F592A^ with 1NMPP1. Statistical analysis performed using a two-way Student’s *t*-test. ** *p*=0.0014. Scale bar: 100 µm. Source data are provided as a Source Data file.

**Supplementary Fig. S11**

Supplementary Fig. S11. Putative axon-to-mesenchymal cell paracrine relationships as derived from single cell Seq datasets. List of top 150 secreted ligands (shown in red) expressed within peptidergic nerve fibers within dorsal root ganglia and their paired receptors (shown in black) as expressed within mesenchymal cells of the HO site. DRG scSeq data derived from ^1^. Mesenchymal cell scSeq derived from cluster 2 of Fig 3. Darker blue indicates higher gene expression.

**Supplementary Fig. S12**

Supplementary Fig. S12. Cell clusters and inflammatory cell changes within HO sites with or without sciatic neurectomy. (a) UMAP showing distribution of cells derived from sham and neurectomy samples. (b) Normalized batch distribution across clusters and samples. (c) UMAP of neutrophils showing distribution of sham and neurectomy samples. (d) Pathway analyses of GO term enrichment for DEGs expressed in sham condition, relative to neurectomy. (e) UMAP of macrophages showing distribution of sham and neurectomy samples. (f) Violin plots showing expression of representative M1 and M2 genes as well as their relative expression (neurectomy vs sham). Cells isolated from N=3 animals per sample.

**Supplementary Fig. S13**

Supplementary Fig. S13. NGF expression with or without sciatic neurectomy. (a) UMAP showing distribution of cells derived from sham and neurectomy samples. (b) UMAP of *Ngf* showing distribution among all samples, along with violin plots below to demonstrate *Ngf* expression among the pericyte/SMC cluster. Cells isolated from N=3 animals per sample.

**Supplementary Fig. S14**

Supplementary Fig. S14. Inflammatory cell frequency with or without sciatic neurectomy. Animals were subjected to HO induction, with or without concomitant proximal sciatic neurectomy. The HO site was examined by flow cytometry for inflammatory cell infiltrates at 7 d post-injury. (a,b) Representative FlowJo plots among sham or sciatic neurectomy groups. From top to bottom: Stratification of CD11b^+^ myeloid cells and Ly6G^+^ neutrophils, stratification of F4/80^+^ mature macrophages, and substratification of Ly6C^low^ and Ly6C^high^ monocytes. (c,d) CD11b^+^ myeloid cells and macrophages among total live cells. (e-h) Quantitative analysis of (e) Ly6C^hi^ monocytes, (f) Ly6C^lo^ monocytes, (g) Ly6G^+^ neutrophils, and (h) F4/80^+^ mature macrophages. For all graphs, each dot represents a single animal, with mean ± 1 SD indicated; N=3 animals for Sham and N=4 animals for Neurectomy. * *p*=0.0003 in comparison to sham surgery, as determined by a two-way Student’s *t*-test. Source data are provided as a Source Data file.

**Supplementary Fig. S15**

Supplementary Fig. S15. Exemplifying the gating strategy for flow cytometry. The FACS gating strategy in this figure correspond to FACS data panels in Supplementary Fig. S14. Cells were gated based on forward scattered cells (FSC) versus side scatter cells (SSC). After this, doublet exclusion was performed both by comparing FSC-height vs FSC-width and SSC-height vs. SSC-width. Propidium Iodide staining determined the live cells and all negative stained cells were gated. Once this was determined, CD11b+ cells was gated. These CD11b cells were then analyzed their expression of granulocyte and myeloid cell markers, Ly6G and Ly6C respectively and gated into Ly6Chi monocytes, ly6Clo monocytes, Ly6C and Ly6G double negative macrophages, and Ly6G positive neutrophils. Macrophages were further analyzed for their expression of macrophage marker F4/80. Unstained controls were used to determine the negative gate.

**Supplementary Fig. S16**

Supplementary Fig. S16. Uncropped scans of the western blots for NGF and pro-NGF. (a) Full scans of NGF and pro-NGF at the injury site at days 0, 3, and 7 by western blot. N=3 animals per group for all assays.

**Supplementary Tables**

Supplementary Table S1: FGF and TGFβ signaling changes following neurectomy. Genes expressed as fold change between neurectomy and sham surgery conditions.

FGF	TGFβ

Gene name	Fold change	Gene name	Fold change

*Fgf2*	2.65560733	*Tgfb3*	0.998459823

*Frs2*	2.421030074	*Tgfbr2*	0.996986403

*Kif16b*	2.344632922	*Crebbp*	0.948062168

*Grb2*	2.236770727	*Tfdp1*	0.894197758

*Sulf1*	2.075733121	*Fstl1*	0.845405569

*Fgf11*	1.927288344	*Cdkn2b*	0.805226351

*Setx*	1.813931627	*Tgfb2*	0.731854314

*Ndst1*	1.693642234	*E2f4*	0.63036003

*Ptpn11*	1.622167302	*Smad3*	0.596400138

*Flrt3*	1.513593644	*Ep300*	0.526724609

*Fgfr2*	1.498914302	*Thbs1*	0.497053331

*Cep57*	1.436266525	*Sp1*	0.426440247

*Iqgap1*	1.380354555	*Rbl1*	0.181963568

*Flrt2*	1.341097101

*Prkd2*	1.221059088

*Fgfr1*	1.193277203

*Rab14*	1.121649666

*Smoc2*	1.106453446

*Dstyk*	1.067869454

Supplementary Table S2. Mouse lines used and primers information.

Mouse lines used

C57BL/6J	Jackson Laboratory, Stock #000664

mT/mG	Jackson Laboratory, Stock #007576

NGF-eGFP	Donated from Kawaja laboratory

*Ngf* ^fl/fl^	Donated from Minichiello laboratory

TrkA^F592A^ 	Donated from Ginty laboratory, Jackson Laboratory, Stock #022362

Primer	Sequence

mT/mG WT Forward	AAGGGAGCTGCAGTGGAGTA

mT/mG WT Reverse	CCGAAAATCTCTGGGAAGTC

mT/mG Mutant Reverse	CGGGCCATTTACCGTAAGTTAT

*Ngf* ^fl/fl^ WT Forward	CACTGCTCTACACCCACCCA

*Ngf* ^fl/fl^ WT Reverse	GGCCGACAAGCAGAAGAACG

*Ngf* ^fl/fl^ Mutant Reverse	AGGCAGCCACAGGGGAATAG

TrkA Forward	AACAGTTTTGAGCATTTTCTATTGTTTAAAAG

TrkA Reverse	CAAAGAAAACAGAAGAAAAATAATACATGAAG

Supplementary Table S3: Antibodies used.

Name	Vendor	Catalog No.	Concentration	Use

Anti-Aggrecan	Abcam	ab3778	1:200	IF

Anti-CD11b	BD Biosciences	562950	1:100	F

Anti-CD45	BioLegend	103144	1:200	IF

Anti-CGRP	Sigma Aldrich	C8198	1:200	IF

Anti-Col2	Abcam	ab185430	1:200	IF

Anti-ColX	Abcam	ab58632	1:200	IF

Anti-FGF2	Santa Cruz	sc-365106	1:100	IF

Anti-F4/80	Abcam	ab204467	1:200	IF

Anti-F4/80	BD Biosciences	746070	1:100	F

Anti-Ly6C	BD Biosciences	553104	1:50	F

Anti-Ly6G	BD Biosciences	551459	1:100	F

Anti-NGF	Abcam	ab6199	1:100	IHC

Anti-NGF	Abcam	ab52918	1:100	WB

Anti-pro-NGF	Santa Cruz	365944	1:100	WB

Anti-Osteocalcin	Abcam	ab93876	1:200	IF

Anti-PDGFRA	Abcam	ab15501	N/A	IF

Anti-PDGFRA	Abcam	ab96569	1:100	IHC

Anti-pERK1/2	Cell Signaling	9101S	1:100	IF

Anti-pTrkA	Invitrogen	PA5-37672	1:200	IF

Anti-PGP9.5	Agilent Tech.	Z511601-2	1:200	IF

Anti-pSmad2	Invitrogen	44-244G	1:100	IF

Anti-S100	Roche Diagnostics	790-2914	N/A	IHC

Anti-SM31	EMD Millipore	NE1022	1:20,000	IHC

Anti-SMA	Abcam	ab7817	1:200	IF

Anti-SOX9	Abcam	ab185230	1:200	IF

Anti-TH	EMD Millipore	AB152	1:200	IF

Anti-TNMD	Abcam	ab203676	1:200	IF

Anti-TUBB3	Abcam	ab18207	1:1500	IF

Anti-Mouse IgG	Vector	MP-5402	1:200	IHC

Anti-rabbit IgG, HRP	Cell Signaling	7074S	1:200	WB

GAPDH	Cell Signaling	5174S	1:1000	WB

Goat Anti-Mouse IgG	Abcam	ab150119	1:200	IF

Goat Anti-Rabbit IgG	Vector	DI-1594	1:200	IF

F: Flow cytometry; IF: Immunofluorescence; IHC: Immunohistochemistry

Supplementary Table S4: Summary demographics of human HO samples.

Age (mean)	Gender (M/F)	Location

33.3 years	Male (50%)	Hip and Leg (75%)

Female (50%)	Other site (25%)
